# Case of Traumatic Tetanus: Administration of Æther

**Published:** 1847-07

**Authors:** H. H. Broughton


*Case of Traumatic Tetanus: Administration of Æther.*—By H. H. Broughton, M. D.—On Saturday, March 20th, I was called at 6 A.M., to Charles Prescott, a miner, residing two miles from this place. I found the left arm completely shattered by a large stone falling on it; he had not lost much blood; his comrade had tied a piece of cord tight round his arm, which completely checked the bleeding. He had walked home a distance of a mile from the shaft where the accident occured. I immediately amputated below the elbow-joint; three arteries required ligatures. He went on exceedingly well for some days. On Wednesday, the 24th, he was most anxious to get up, and on the wound being dressed, union had taken place by the first intention. On Saturday, the 27th, I found him sitting up and dressed; he said he was quite tired of bed. In the evening he sent down, stating he had taken cold. He had some aperient medicine sent him.

On Sunday the symptoms of tetanus became marked, there was considerable rigidity of the muscles of the neck and jaw, and difficulty in swallowing. He had an enema with turpentine, calomel, and an active aperient, which soon operated. The symptoms continuing, he had thirty minims of liquor opii sedativus, every hour, and belladonna to the neck and jaws. He was perfectly under the influence of opium, but without any remission of the symptoms of opisthotonos.

Monday evening. The spasms now most violent. Half a drachm of æther, added to each dose of opium, and calomel freely given through the night.

Tuesday morning. No better, but decidedly worse. We now determined to try æther. This was administered by means of Boott's apparatus. He was soon under its influence, and immediately all contraction and spasm ceased, and he got into a most comfortable sleep, which lasted full ten minutes. As soon as he became sensible the spasm and contraction returned, but scarcely so violent. He again enhaled the æther with the same result. He had it a third time; it again relieved him, and he was left asleep, and on visiting him I found he could open his mouth better. He was most anxious to have it again, but a violent spasm came on before I could get it to him, followed by another, and he immediately sank.—*Ibid.*

